# Extraversion differentiates between model-based and model-free strategies in a reinforcement learning task

**DOI:** 10.3389/fnhum.2013.00525

**Published:** 2013-09-03

**Authors:** Anya Skatova, Patricia A. Chan, Nathaniel D. Daw

**Affiliations:** ^1^The School of Psychology, University of NottinghamNottingham, UK; ^2^Horizon Digital Economy Research, University of NottinghamNottingham, UK; ^3^Department of Psychology, Center for Neural Science, New York UniversityNew York, NY, USA

**Keywords:** extraversion, dopamine, reinforcement learning, personality, decision-making

## Abstract

Prominent computational models describe a neural mechanism for learning from reward prediction errors, and it has been suggested that variations in this mechanism are reflected in personality factors such as trait extraversion. However, although trait extraversion has been linked to improved reward learning, it is not yet known whether this relationship is selective for the particular computational strategy associated with error-driven learning, known as model-free reinforcement learning, vs. another strategy, model-based learning, which the brain is also known to employ. In the present study we test this relationship by examining whether humans' scores on an extraversion scale predict individual differences in the balance between model-based and model-free learning strategies in a sequentially structured decision task designed to distinguish between them. In previous studies with this task, participants have shown a combination of both types of learning, but with substantial individual variation in the balance between them. In the current study, extraversion predicted worse behavior across both sorts of learning. However, the hypothesis that extraverts would be selectively better at model-free reinforcement learning held up among a subset of the more engaged participants, and overall, higher task engagement was associated with a more selective pattern by which extraversion predicted better model-free learning. The findings indicate a relationship between a broad personality orientation and detailed computational learning mechanisms. Results like those in the present study suggest an intriguing and rich relationship between core neuro-computational mechanisms and broader life orientations and outcomes.

## Introduction

It is widely hypothesized that the brain learns from rewards using a prediction error-driven learning rule (Bush and Mosteller, 1953; Rescorla and Wagner, 1972). Prediction errors are thought to drive learning on trial-and-error decision tasks by reinforcing successful actions (a scheme dating back to Thorndike, 1911), and have reliable neural correlates, notably in the firing of neurons containing the neuromodulator dopamine (Houk et al., 1995; Schultz et al., 1997) and in blood oxygenation signals recorded in human functional imaging studies (McClure et al., 2003; O'Doherty et al., 2003). In humans, this mechanism's contribution to learning is evidenced by numerous links between learning performance, neural signatures of reward prediction errors, and/or dopaminergic action (Frank et al., 2004; Pessiglione et al., 2007; Schonberg et al., 2007, 2010; Cools et al., 2009; Voon et al., 2010).

It has also long been suggested that individual differences in reward processing mechanisms such as this one contribute to variations in personality. In an influential review, Depue and Collins (1999) argued for an association between variation in incentive motivation and extraversion, and suggested that this association might be rooted in a dopaminergic mechanism. This work inspired a line of research establishing that extraversion and related personality traits (impulsivity, reward sensitivity, approach motivation, and the behavioral activation system) have links with reward processing (Smillie, 2013). For instance, extraversion and its relatives predict behavioral performance, specifically response bias for rewarded alternatives, on laboratory learning tasks (Corr et al., 1997; Pickering, 2004; Smillie et al., 2007), and more real-world reward-driven behaviors such as eating disorders and drug abuse (Dawe et al., 2004; Dawe and Loxton, 2004). Also, speaking to the relationship between these functions and underlying neural mechanisms, extraversion and similar measures are associated with neural activity related to prediction errors and at dopamine targets (Cohen et al., 2005; Smillie et al., 2011), and genetic polymorphisms related to dopamine expression (Smillie et al., 2010).

Altogether, these experiments suggest that extraversion is associated with reward processing, potentially reflecting variation in a reward prediction error-based learning mechanism (Cohen, 2007; Pickering and Smillie, 2008). However, it has recently become appreciated that such error-driven reinforcement is not exclusive, but instead that the brain contains multiple distinct or even competing pathways for learning from reward (Dickinson and Balleine, 2003; Daw et al., 2005; Rangel et al., 2008). One prominent computational version of this idea (Daw et al., 2005) suggests that the *model-free* reinforcement strategies traditionally associated with error-driven updating are accompanied by an additional system for *model-based* reinforcement learning. Whereas a model-free strategy essentially consists of learning to repeat rewarded actions, model-based algorithms learn a map or model of the structure of the task, and use it to evaluate candidate actions more deliberatively by mental simulations of their consequences. Model-based learning does not rely on reward prediction errors (Gläscher et al., 2010), but it does verifiably contribute to human and even rodent behavior (Dickinson and Balleine, 2003; Daw et al., 2011). The classic example of the distinction between model-free and model-based reinforcement learning is the notion that a rat, when pressing a lever that delivers food, might be doing so for at least two reasons. The first reason, associated with model-free RL, is that the rat has learned that pressing the lever is desirable, because previous leverpresses have been rewarded. The model-based alternative is that the rat might have learned that the lever delivers food, and that the food is desirable, and from this “model” of the action's specific consequences, the rat can conclude that pressing the lever is valuable. This distinction can be tested (an idea going back to Tolman, e.g., Tolman et al., 1946) by examining how subjects adjust their behavior to changes in their goals or the task contingencies: in a way consistent with the model-free reinforcement principle of repeating previously successful actions, or instead in a way that reflects the use of a model of the task contingencies to re-evaluate actions in terms of the newly changed circumstance. In the example of a rat leverpressing, one may ask whether the rat continues to press the lever even if the food is no longer desirable (e.g., if the rat is fed to satiety; Dickinson and Balleine, 2003), as is predicted by model-free but not model-based learning.

Importantly, most laboratory reward tasks do not contain such a manipulation to differentiate which (or what mixture) of these two mechanisms supports learning behavior. Instead, behavior is typically ambiguous as to the underlying learning strategy, and what is apparently the same behavior may reflect different mixtures of their influences in different subjects or circumstances (Dickinson and Balleine, 2003; Daw et al., 2011). In particular, the behavioral tasks so far used to investigate a link between reward learning and extraversion do not establish whether the reward learning behavior is consistent with having been produced by (model-free) reward prediction errors, unconfounded from model-based mechanisms.

This suggests the hypothesis that we test in our present study: that trait extraversion will relate selectively to model-free rather than model-based learning. Such an idea is supported by the links between extraversion, prediction errors, and dopamine, in light of the role of prediction errors in model-free learning. Alternatively, extraversion might not be selective in this manner. For instance, there is some evidence that neural prediction errors (Daw et al., 2011) and dopaminergically mediated learning (Wunderlich et al., 2012) are themselves not entirely selective for model-free learning.

The model-based vs. model-free distinction comes from machine learning (e.g., Sutton and Barto, 1998) and relates most closely to previous theoretical ideas in animal learning and computational neuroscience (Daw et al., 2005). However, this computational distinction may also be related to other dual-process theories, notably in human cognitive psychology and cognitive neuroscience where researchers have long distinguished between processes that are variously described as automatic, procedural, or incremental vs. deliberative, declarative, or rule-based (e.g., Sloman, 1996; Ashby and Maddox, 2005). In this respect, another previous result suggesting the present hypothesis is a study (Pickering, 2004) that argued that extraversion was selectively linked to procedural rather than rule-based learning (which may parallel model-free vs. model-based; Otto et al., 2013). Specifically, Pickering (2004) reported that in experiments with category learning tasks, performance on conditions requiring integrating information from various stimulus dimensions was linked to extraversion. Conversely, performance on paired-associate learning tasks was not linked to extraversion. Although these tasks clearly differ on many dimensions, one salient difference is that the former tasks are believed to promote incremental learning and the latter to promote rule-based or memorization-based solution.

Thus, Pickering's (2004) comparison between the tasks is suggestive, but one advantage of the model-based vs. model-free dichotomy is that the contributions of both processes can be quantified and compared on even ground, in the context of a single task that simultaneously engages both. The present, computational view also substantially refines the more cognitive one, by specifying a quantitative, computational mechanism and situating it in the context of a body of work on animal learning and its mechanistic neural substrates.

Note that whereas in the human literature, the status of learning as explicit vs. implicit has been taken as a key or even defining characteristic of the two processes, the model-based vs. model-free distinction is defined operationally, in terms of different learning rules, and makes no particular claim about conscious access. However, model-free learning resists (while model-based learning is obliterated by) dual-task interference (Otto et al., 2013) in a manner similar to other signature implicit learning tasks (Nissen and Bullemer, 1987; See Daw and Shohamy, 2008; Daw and O'Doherty, 2013; Otto et al., 2013; for more discussion of the relationships between different dual-process theories).

In the present study we attempt more finely to dissect the relationship between trait extraversion and learning from reward by comparing extraversion to behavior on a two-step decision task which is designed to distinguish model-based from model-free learning (Daw et al., 2011). The logic of the task, discussed in more detail below, is that the different learning rules predict different patterns of trial-to-trial adjustment of choice preferences in light of the new information given to the participant by each trial's outcome. By examining patterns of switching in this multistep task (where two choices are made in sequence), it is possible to distinguish retrospective, model-free mechanisms (repeating previously successful actions) from more prospective, model-based learning, which evaluates options in terms of their expected consequences at the next step. In previous studies with this task, participants were shown to use a combination of both model-based and model-free decision-making mechanisms, but with substantial individual variation in the balance between them. If the previously reported facilitation of reward learning in extraverts were selective for model-free behavior, this would provide further support for the nexus of function that ties together extraversion and the error-driven learning mechanism.

## Methods

### Participants

We tested two subsamples of participants. All participants were recruited through a New York University message board. Informed consent was obtained from all participants. The first subsample was collected from October to November 2009, *N* = 48 (*M*_age_ = 21.7, 68% female). The second subsample was collected from September to October 2012, *N* = 50 (*M*_age_ = 24.8, 64% female). There were no significant differences between the two subsamples in terms of age [*t*_(95)_ = −1.83, *p* = 0.07] and gender [χ^2^_(1, *N* = 97)_ = 0.182, *p* = 0.913]. The experimental procedures for both subsamples were identical, with the following exceptions. The first subsample completed 350 trials of the decision-making task, with inter-state and inter-trial intervals of 500 and 300 ms, respectively. The second subsample completed 300 trials of the decision-making task, and the inter-state and inter-trial intervals were 1500 and 1000 ms. (The changes were intended to improve the participants' quality of decisions, as the longer time of the overall procedure, shorter inter-stimuli interval, and inter-trial interval might have imposed a greater cognitive demand on the participants in the first sample.)

One participant was excluded from all analyses because a complete dataset was not obtained due to a software crash. This left 97 participants for the reported results.

### Measures

Participants began by filling out the extraversion subsection of the EPQ-R questionnaire (Eysenck et al., 1985) via a computer. Next, participants completed 350 or 300 trials (see further explanations in the Participants subsection) of the two-step decision task (Daw et al., 2011). Halfway through the trials, participants took a short break.

**Figure 1:**
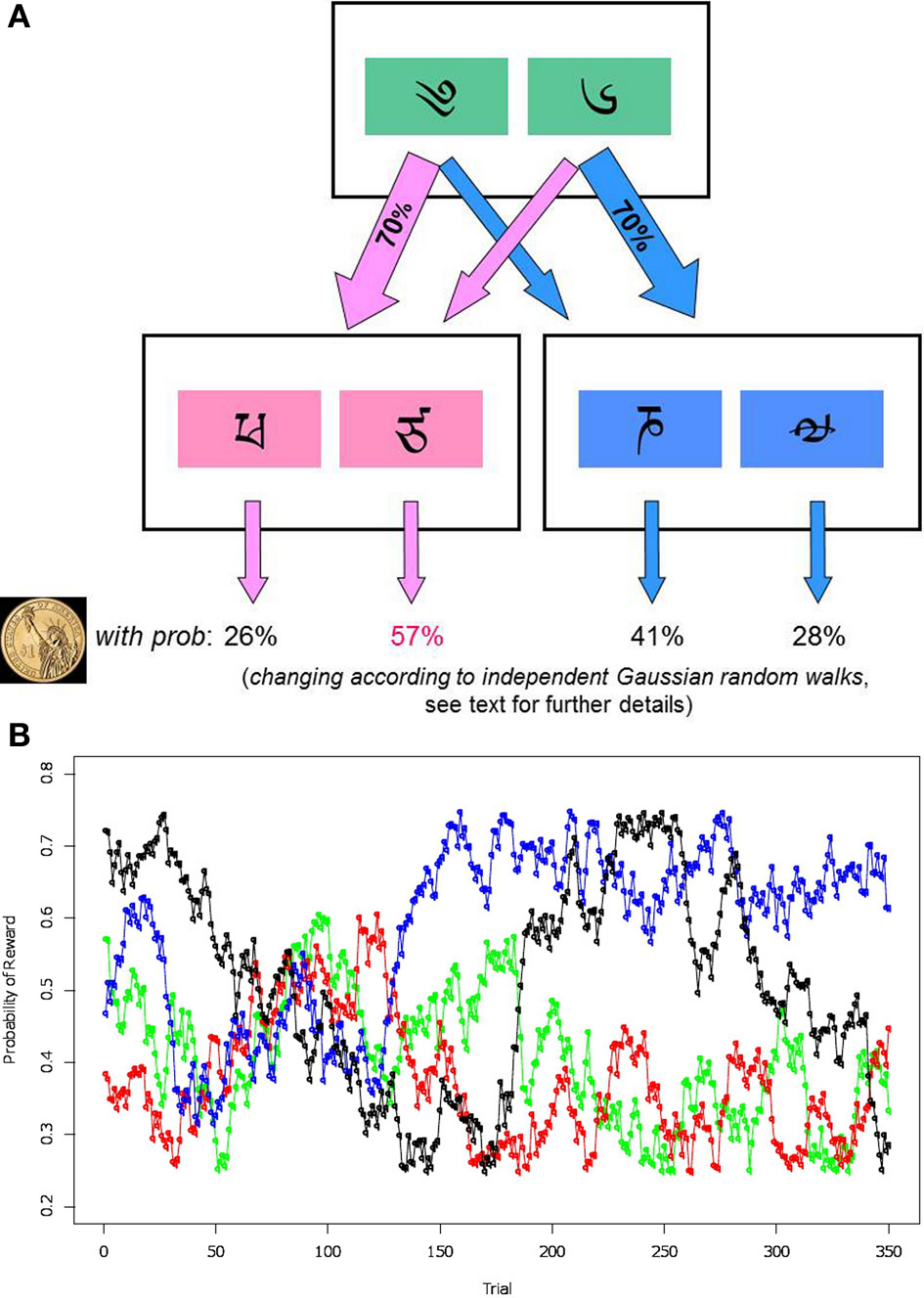
**(A)** Schematic representation of the sequential task. Participants are first presented with two first-stage boxes. Each of the boxes (right or left) has a fixed probability of leading to one of the two pairs of second-stage boxes in 70% of cases, and to another pair in 30% of cases. After participants make a first-stage decision, they need to make a second choice, between the second-stage boxes. This leads them to receiving a reward, or none (depicted by a dollar image), based on which second-stage box they choose. **(B)** An example of reward probabilities when choosing the second-stage box. Lines of four different colors represent how probabilities change for four possible second-stage boxes. To encourage participants to learn continually, the reward probabilities diffuse according to independent Gaussian random walks.

The two-step decision-making task was designed to measure the extent to which each individual participant relies on model-based and model-free learning strategies. The task details were as described by Daw et al. (2011), with the exception that subjects completed more trials separated by shorter inter-event breaks. In the task, participants made a series of two decisions on each trial, and were then given either a single monetary reward, or nothing. The first decision made (i.e., the choice at the first stage) affected the options for the second-stage decision; see the schematic representation of the task in Figure 1A.

Specifically, on the first stage, participants were presented with two boxes labeled by Tibetan characters (green boxes, Figure 1A). Each box led probabilistically to either of two pairs of second-stage boxes (pink and blue boxes, Figure 1A). The two possible second-stage alternatives consisted of another pair of boxes represented by new Tibetan characters (pink and blue boxes, Figure 1A). Which of these two pairs of boxes was presented was determined, stochastically, by the first-stage decision. Each option in the second stage was associated with a different probability of winning a monetary reward (vs. nothing) when chosen. To encourage ongoing learning, the chances of payoff associated with the four possible second-stage options were changed slowly and independently throughout the task, according to independent Gaussian random walks. (At each step, each reward probability was perturbed by adding Gaussian noise with mean zero and *SD* = 0.025, with reflecting boundary conditions at 25 and 75%. Figure 1B depicts an example of how the win probabilities changed for all four boxes.) The goal of the participants was therefore to earn the most money by tracking which second-stage box was currently most rewarding, and by choosing the first-stage box most likely to lead to it.

The probabilistic coupling between first-stage choices and second-stage options was as follows. Seventy percent of the time (a “common” transition) the choice of each of the first stage boxes led to an associated pair of second-stage boxes. This relationship remained the same over the course of the experiment. The other 30% of the time, however, each first-stage choice led to the other second-stage box not usually associated with it (a “rare” transition). For example, if a participant chose the left green box (Figure 1A), in 70% of cases they would experience a common transition and see on stage two a pink pair of boxes, while in 30% of cases they would encounter a rare transition and get the blue pair of boxes. The common/rare transition probabilities were reversed for the right first-stage green box. At the conclusion of the experiment, task winnings were paid in real money at a fractional rate.

### Analysis strategy

Model-free and model-based RL approaches have different consequences for trial-by-trial adjustments in action preferences in light of the events on each trial, which can be assessed by regressing recent rewards on choices (Daw et al., 2011; Wunderlich et al., 2012; Otto et al., 2013). To assess the extent to which a participant relies on a model-based or model-free strategy, we evaluated the effect of events on each trial (trial *n*) of the second-stage choice on the subsequent trial (trial *n* + 1). The two key events on trial *n* were whether or not a reward was received, and whether this occurred after a common or rare transition to the second stage state, given the first-stage choice on trial *n*. We evaluated the impact of these events on the chance of repeating the same first-stage choice on trial *n* + 1.

The logic for this approach (see also Daw et al., 2011) was that model-free RL (e.g., the TD-λ algorithm for λ > 0) would tend to repeat a choice that results in reward regardless of in which state that reward occurred, predicting a positive main effect of reward. Model-based RL instead evaluates first-stage actions in terms of the second-stage alternatives they tend to lead to; for this reason, the effect of a reward at the first stage depends in which pair of boxes it was received, and an interaction of reward by transition (common or rare) is predicted. For instance, consider a trial in which a subject chooses the left green box at the first stage, but received the rare (blue) boxes, and was ultimately rewarded for their choice. A model-free learner will be more likely to repeat the first-stage choice following this trial (since it was ultimately rewarded); a model-based learner will, conversely, be more likely to choose the other first-stage choice (since this is the one that is more likely to lead to the blue boxes where the reward was received).

According to this logic, we take the main effect of reward as an index of model-free learning (where larger positive effects indicate more model-free switching) and the reward by transition interaction as an indication of model-based learning (where larger positive effects indicate more model-based switching, since the interaction inverts the sign of the reward effect for rare transitions, which are coded as −1). In previous studies using this task (Daw et al., 2011; Wunderlich et al., 2012; Otto et al., 2013) participants have exhibited a mixture of both effects.

We analyzed these effects using multilevel logistic regression, using the lme4 package (Bates et al., 2012) in the R statistical environment (R Development Core Team, 2011). For each trial after the first, the regression predicted the probability of staying with the previously chosen first stage option (vs. switching) as a function of four population-level predictor variables (which were in later analyses each further interacted with one or two between-subject covariates). At the level of each subject, the basic model was a 2 × 2 factorial model with factors of reward and transition. This gives rise to four predictors: (1) whether, on the preceding trial, the subject received a reward (1 if rewarded, −1 if unrewarded), (2) whether, the transition from the first-stage to the second-stage choice was common or rare probability (1 if common, −1 if rare), (3) the multiplicative interaction of the reward and transition regressors; (4) an intercept term, which reflects a tendency to perseverate or switch regardless of the events in the task, e.g., regardless whether the previous option was rewarded or not. At the group level, these four effects were all taken as random effects, i.e., each instantiated once per subject from a population distribution. As described below, we also included group-level predictors, such as extraversion, interacted with these factors. Note that only two of these effects—the main effect of reward and its interaction with the transition type—are relevant to the learning model, and only one (the main effect of reward) to our particular hypothesis about model-free learning in this study. The others are included in the model to ensure a more balanced, factorial design.

To assess whether model-based and model-free learning effects covaried with extraversion, the four explanatory variables were each interacted, across subjects, with the participants' extraversion scores. This produced four more group-level coefficients (the main effect of extraversion, its two-way interactions with reward and transition, and the three-way interaction between all factors) characterizing to what extent each of the baseline model parameters changed, across subjects, as a function of their extraversion scores. The extraversion scores were converted to Z-scores prior to being entered in the analysis. Again, our main hypothesis concerns the relationship between model-free RL and extraversion (the extraversion by reward effect), with that for model-based RL (the three-way interaction between reward, transition, and extraversion) also of interest, but we estimate a full factorial model with all interactions to ensure that our results are specific to the hypothesized interaction unconfounded by the other, unhypothesized possibilities. In designing and carrying out these analyses, we were guided by Gelman and Hill (Gelman and Hill, 2007; see also Gelman et al., 2003), who tend to advocate against excluding potential explanatory variables, especially in the context of a multilevel model.

Finally, to examine whether the relationship between extraversion and RL task performance was affected or obscured by between-participant variations in task motivation or engagement, we defined a measure of task responsiveness (“engagement”). This overall sensitivity to events in the RL task was measured by fitting the logistic regression described before to each participant's choices individually. At the individual level, this model involves four effects of intercept, reward, transition, and reward by transition, but not the between-subject terms involving interactions with extraversion. We scored each participant's overall sensitivity to the RL task by subtracting the model's deviance from the deviance of a reduced logistic regression model containing only the intercept, i.e., an average tendency to stay or switch but no learning effects at all. The logic of this measure was to characterize the extent to which subjects' choices were responsive to the events in each trial, without assuming either a model-based or model-free form for this dependence. The difference of deviances is a measure of the relative fit of the two models to the data—thus, measuring how much better the choices are explained by assuming the subject adjusts their preferences in light of each trial's outcome according to any combination of the factors of reward and transition, vs. responding at random or with constant preferences. (Specifically, this measure is the test statistic for the likelihood ratio test comparing these models, and is related to the approximate log Bayes factor between them; Kass and Raftery, 1995). In order to be able to obtain unbiased estimates of the relationship between extraversion, the engagement score, and learning strategy, we defined the engagement score using fits to only odd-numbered trials, while we tested the relationship between variables of interest on only the even-numbered trials. This ensured that engagement was defined on a different set of data than those on which its effects were tested; avoiding any bias that otherwise might arise from defining and testing the effect on the same data subset.

We used this engagement score both to define a subgroup of highly responsive participants (the top 20% on this score, across both subsamples) for separate analysis, and also entered it (Z-scored) as a covariate in an additional version of the RL regression, interacted with the basic RL effects and their interactions with extraversion to produce eight more predictors. (Again, the hypothesis concerns the three-way interaction of reward by extraversion by engagement, but we include all factorial interactions to ensure the interpretability of this result.)

The R formulas for these models were:

stay ~ trans * rew * extra + (1 + trans * rew | subID)

and

stay ~ trans * rew * extra * engage + (1 + trans * rew | subID)

which were estimated using the “glmer” function with family = binomial.

## Results

**Figure 2:**
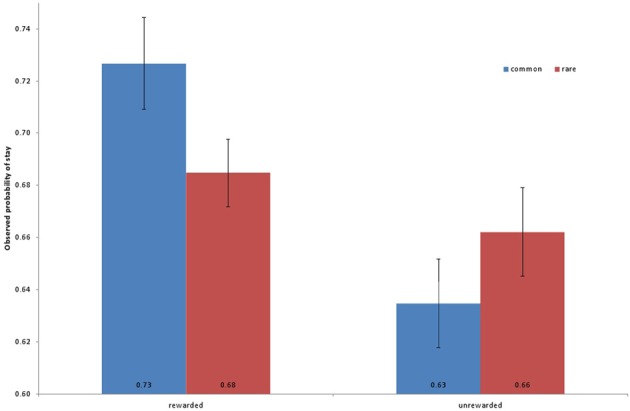
**Observed frequencies of repeating a first-stage choice in the second stage (“stay probability”) as a function of whether the previous trial's choice was rewarded (vs. not) and the transition was common (i.e., the more likely one, given the first-stage choice) or rare.** Frequencies are averaged across participants; on average, participants display evidence for both model-free learning (main effect of reward) and model-based (its interaction with transition).

Subjects completed a two-stage decision task (Daw et al., 2011). They failed to complete a small fraction of trials (average number of missed trials, 1.4%, ± 0.53 SEM) due to response time limits. They received reward for, on average, 50.8% (±0.44 SEM) of their completed trials. Figure 2 depicts the observed frequency of staying with a top-stage choice as a function of the previous trial's reward and transition, averaged across the sample.

To examine individual differences in subjects' trial-by-trial learning strategies in the RL task, we used a mixed effects logistic regression to explain each trial's first-stage choice in terms of the events on the previous trial (Table 1, Daw et al., 2011). As expected, evidence for both model-free and model-based influences on choices was observed at the group level, but with individual variability in their degree. In particular, the reward on a trial significantly predicted the subsequent choice (a marker for model-free RL, see Methods; β = 0.198, *Z* = 7.36, *p* < 0.001), and the interaction of the reward effect with transition (whether the reward was received after a common or rare state transition, indicative of model-based RL) was also positive (β = 0.132, *Z* = 6.19, *p* < 0.001).

**Table 1:**
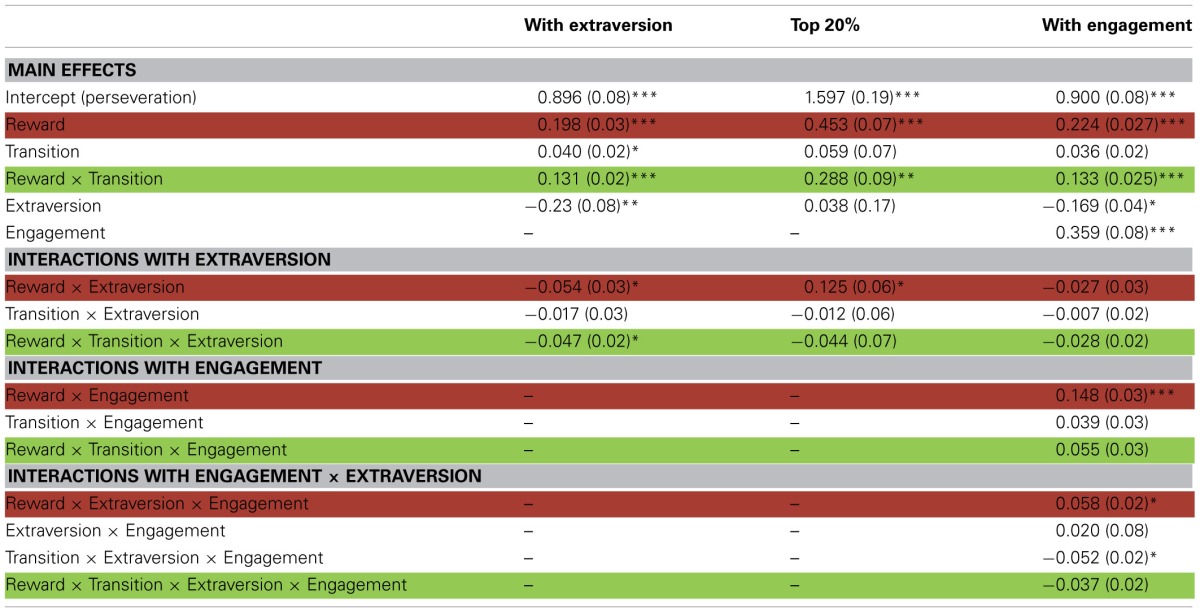
**RL and extraversion effects for the overall sample of 97 participants, top 20%, and with engagement index: Beta coefficient estimates with standard errors from three mixed effects logistic regression analyses**.

^***^p < 0.001, ^**^p < 0.01, ^*^p < 0.05;

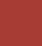
 *model-free*, 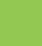 *model-based*.

As the data for this study were collected in two subsamples with some variations in task timing (see Methods) we tested for differences between the groups by including an indicator variable for subsample interaction with all effects in the regression. The only such effect that reached significance was the main effect of subsample, indicating that participants in the first subsample tended to switch more often than participants in the second subsample (β = 0.22, *Z* = 2.62, *p* = 0.008). Seeing no differences with respect to the behaviors of interest, we conducted the remaining analyses in this study on data from the combined group of 97 participants.

We examined the relationship between scores on the extraversion scale and RL task performance. The mean score on the extraversion scale for the first subsample was 16.52 with a standard deviation of 4.69, and α = 0.85. The mean score for the second subsample was 15.2 with a standard deviation of 5.06, and α = 0.85. There was no significant difference in extraversion scores between the two subsamples: *t*_(96)_ = −1.34, *p* = 0.18.

**Figure 3:**
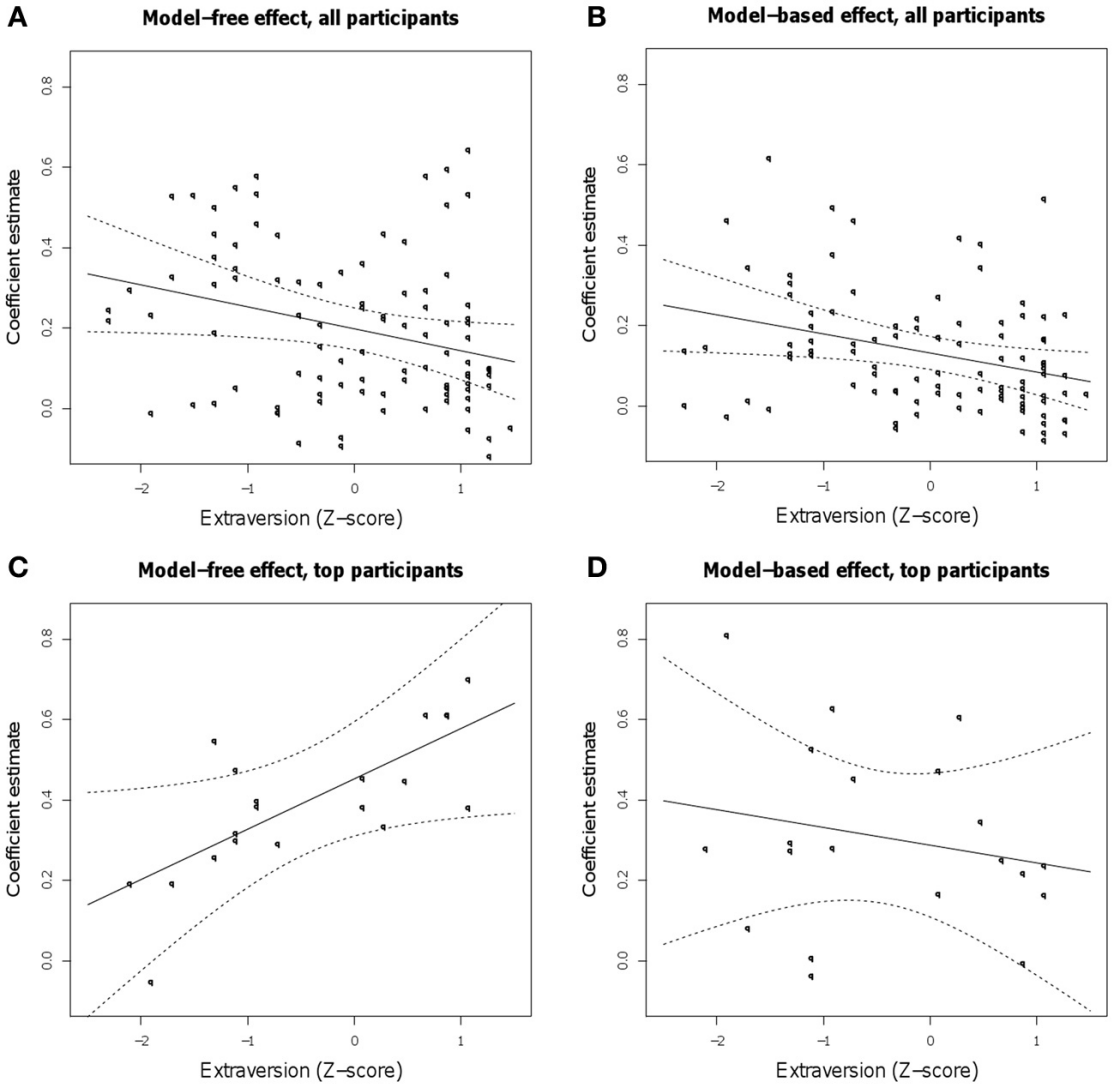
**Estimated linear relationship between extraversion and the size of the model-free and model-based learning effects [the regression coefficients for the main effect of reward (A) and its interaction with the transition type (B), respectively] in the full population and in the top 20% of subjects as measured by task engagement (C and D).** Shown are the group-level linear effect, with 95% confidence curves, over points representing each participant's estimated individual effect, conditional on the group level-estimate.

Extraversion scores were included as a covariate in the regression on the RL task. Here, positive interactions with reward or reward by transition would indicate better model-free or model-based RL (respectively) for subjects with higher extraversion scores. This factor interacted significantly with our indicators for both model-free and model-based learning, with higher extraversion indicating a decreased influence of both strategies (Figures 3A,B). In particular, the interaction of extraversion with reward (model-free) was negative (*Z* = −2.04, *p* = 0.041), and the three-way interaction of extraversion, reward, and transition (model-based) was significantly negative (*Z* = −2.25, *p* = 0.024). Thus, personality scores did not have the hypothesized selective effect on model-free learning, nor even the previously reported facilitatory impact on any sort of reward learning; instead, higher extraversion predicted generally poorer performance on both sorts of RL. In addition, there was a main effect of extraversion: high extraverts tended to switch more than participants low in extraversion (*Z* = −2.71, *p* = 0.007).

These results raise the possibility that a general disengagement from or unresponsiveness to the experimental task, associated with extraversion, was masking more selective influences of extraversion on learning strategy. Such a generalized relationship between subject performance and extraversion is consistent with reports that under some conditions trait extraversion tends to predict less accurate responses and faster reaction times on certain tasks (Matthews and Gilliland, 1999; Wacker et al., 2006). To examine this possibility, we first repeated our analysis in a sample of 20% of participants (*N* = 20) chosen for the best responsiveness to the RL task (a subject engagement measure measuring the difference in each subject's model fit between the learning model and a null model, with a higher score reflecting higher task engagement; see Methods). For this and the subsequent analysis, we defined task engagement based on the performance on odd trials, and tested behavior by fitting a model to the remaining, even trials. Consistent with the previous finding that higher extraversion predicted worse performance on the RL task, engagement and extraversion trended toward being negatively correlated [*r* = −0.19, *t*_(95)_ = −1.89, *p* = 0.062], though this relationship was not significant. Similarly, the extraversion scores for the 20% high performing subjects were on average lower than those for the other 80% [–0.44 vs. 0.11; *t*_(28)_ = −2.15, *p* < 0.05], though well-distributed throughout the range.

Within the subgroup of high performing subjects, higher extraversion scores predicted better learning from reward in a model-free fashion (positive extraversion by reward interaction, Z = 2.02, *p* = 0.0432; Table 1, Figure 3C) together with no significant relationship to model-based learning (*Z* = −0.559, *p* > 0.5; Figure 3D), a result which was in line with the original hypothesis.

Finally, to investigate whether it is indeed the case that in the full sample, the association between extraversion and RL strategy depends on a participant's overall task engagement, we repeated the same regression analysis as previously, but additionally testing the interaction of all factors with the engagement measure (again, defined on a non-overlapping subset of trials to avoid bias). Here, a positive three- or four-way interaction (engagement by extraversion by reward or engagement by extraversion by reward by transition) would indicate a pattern whereby the relationship between extraversion and model free (or, respectively, model-based) learning became more facilitatory for more engaged participants. Indeed, the engagement measure interacted positively with the association between extraversion and model-free learning (*Z* = 2.11, *p* = 0.0344) and not significantly with the association between extraversion and model-based learning (*Z* = −1.436, *p* = 0.1511). Directly comparing these effects using a linear contrast, we verified that the relationship between engagement, extraversion and model-free learning was larger than that for model-based learning (i.e., the effect of engagement is specific to model-free learning; χ^2^(1, *N* = 97) = 5.75, *p* = 0.01). Thus, to the extent that a participant was more responsive to the task, this was selectively associated with a stronger positive coupling between extraversion and model-free learning.

## Discussion

Previous studies have shown that extraversion is associated with enhanced reward sensitivity (Pickering, 2004; Smillie et al., 2011). In the current study, we aimed to revisit and refine this association. We assessed the relationship between extraversion and individual differences in the specific, model-free learning strategy most commonly associated with learning from reinforcement in the brain, by using a reinforcement learning task that distinguishes this mechanism from more deliberative, model-based learning that typically confounds it. Contrary to the hypothesis, we found that overall extraversion was associated with poorer reinforcement learning on both model-based and model-free dimensions, apparently reflecting poor task engagement. However, the hypothesis that extraverts would be better at model-free RL did hold up in a subset of the more engaged participants, and accordingly, across the full group, higher task engagement was associated (on a different subset of trials) with a shift toward the expected pattern, by which extraversion selectively promoted model-free RL.

At least among the more engaged participants, then, these results demonstrate a relationship between a broad personality orientation and a detailed computational learning mechanism. Moreover, although we are manifestly not in a position to infer any causation and, in this study, did not measure any observables directly related to dopaminergic function, these findings are consistent with other suggestions that both of these aspects of behavior may arise due to a common dopaminergic cause. They also fit well with and sharpen previous results using category learning, which showed a positive association between extraversion and incremental, but not rule-based learning (Pickering, 2004).

At the same time, given such previous reports linking extraversion to improved reward learning, the unhypothesized relationship in our full sample between extraversion and more generically *worse* reinforcement learning performance is puzzling. Especially combined with increased alternation between options from trial to trial in extraverts, the pattern of their choices, which was more weakly sensitive to reward feedback suggests that these participants were simply less engaged with or responsive to the task. It may be that this complex, multistep learning task is more cognitively demanding and/or less engaging than others previously tested with extraversion, promoting a previously subtler tendency among extraverts to disengage. Hints of a tendency toward impatient or careless performance among extraverts might also be seen in previous findings that under certain conditions extraverts tend to be less vigilant and attentive than introverts (Matthews and Gilliland, 1999).

Testing this interpretation remains an important issue for future work. It should be possible to modulate task difficulty (e.g., by manipulating the speed at which options change) within the present task, and/or compare the sequential decision task to traditional one-step tasks so as to examine whether extraverts are sensitive to harder task demands.

In any case, it does appear that overall poor task performance among extraverts in our sample masked the more specific relationship by which (to the extent participants were engaged in the task) extraversion promoted model-free learning. It is also possible that task attentiveness, operationalized by our measure of participant engagement, was capturing the contribution of some additional competing or interacting cognitive or motivational process, which we did not account for in our study. For example, other researchers have pointed out that neuroticism and associated traits can have effects (in some cases, interacting with extraversion) on the performance in learning tasks (Pickering et al., 1995). Further, in tasks similar or identical to the one used here low working memory capacity (Gershman et al., 2012) or concurrent working memory demand (Otto et al., 2013) biases individuals away from model-based choices, leading them to rely on model-free strategy. Future studies can examine cognitive load and personality traits on task performance in RL to investigate whether these other factors mediate or interact with the present results.

Taken together with evidence linking individual differences in pharmacological manipulations of dopaminergic function to performance on learning tasks, our results may indirectly support the idea that individual differences in dopamine are associated with trait extraversion. For instance, individuals with higher baseline synthesis in the striatum demonstrated better learning from rewards in a reversal learning task (Cools et al., 2009), and there are several reports of reward learning deficits in Parkinson's disease that are remediated by dopamine replacement medication (Frank et al., 2004; Bodi et al., 2009). Both in Parkinson's patients (Voon et al., 2010) and healthy participants (Pessiglione et al., 2006), the dopamine precursor L-Dopa promotes learning from reward and reward prediction error-related striatal activity in an instrumental learning task. One note of caution for interpreting the current study's results in dopaminergic terms is that a recent attempt to test the widely hypothesized linkage of dopamine, specifically, to model-free learning using the same task we use here (Wunderlich et al., 2012) instead reported that L-Dopa, paradoxically, promoted model-based over model-free reinforcement learning. However, (as discussed in that report) there are several interpretations of such result consistent with the otherwise substantial evidence that dopamine subserves a prediction error for model-free temporal difference learning.

Finally, our findings highlight something of a disconnect between the way reward processing in extraversion is viewed through the lens of personality research (which is typically focused on broader life trends and higher level decisions) vs. neuroscientific research (which is typically focused on neural underpinnings of short-term choices in laboratory tasks). In personality psychology there is often a sense that extraversion is beneficial (e.g., associated with positive life outcomes Herringer, 1998; Ryan and Deci, 2001; Williams et al., 2004; Jylhä et al., 2009), whereas the specific model-free learning mechanism linked to extraversion here and to dopamine generally is not necessarily so benign. Notably it is a prominent hypothesis that a dominance of model-free over model-based decisions (or “habitual” over “goal directed” processes) contributes to disorders of compulsion, such as drug abuse (Everitt and Robbins, 2005; Redish et al., 2008). Some complementary results are reported in the personality research field. For instance, Francis (1997) found associations between extraversion and positive attitudes toward substance use in a large sample of pupils between 13 and 15 years old. Further, extraversion positively predicted the number of drugs tried by adolescents whose parents were alcoholics (Conner et al., 2010) and traffic offending in young males (Renner and Anderle, 2000). Although such tentative evidence exists, the possibility that extraversion can predict negative life outcomes and the mechanisms by which it may do so remain largely under-investigated. Results like those in the present study suggest an intriguing and rich relationship between core neuro-computational mechanisms and broader life orientations and outcomes.

### Conflict of interest statement

The authors declare that the research was conducted in the absence of any commercial or financial relationships that could be construed as a potential conflict of interest.
